# Metabolic score for insulin resistance predicts major adverse cardiovascular event in premature coronary artery disease

**DOI:** 10.18632/aging.205710

**Published:** 2024-04-01

**Authors:** Dachuan Guo, Chong Zhang, Mingyan Zhang, Zhenguo Wu, Xiaoyu Liu, Yerui Zhang, Li Liu, Meili Sun, Jianmin Yang

**Affiliations:** 1 National Key Laboratory for Innovation and Transformation of Luobing Theory, The Key Laboratory of Cardiovascular Remodeling and Function Research, Chinese Ministry of Education, Chinese National Health Commission and Chinese Academy of Medical Sciences, Department of Cardiology, Qilu Hospital of Shandong University, Jinan, China; 2 Department of Internal Medicine, Jiyang District Traditional Chinese Medicine Hospital, Jinan, China; 3 Department of Oncology, Jinan Central Hospital, Shandong University, Jinan, China; 4 Department of Cardiology, People Hospital of Huantai County, Zibo, China; 5 Department of Oncology, Central Hospital Affiliated to Shandong First Medical University, Jinan, China; 6 Cardiovascular Disease Research Center of Shandong First Medical University, Central Hospital Affiliated to Shandong First Medical University, Jinan, China

**Keywords:** premature coronary artery disease, insulin resistance, major adverse cardiovascular events, prognosis, metabolic score for insulin resistance

## Abstract

Background: The Metabolic Score for Insulin Resistance (METS-IR) index serves as a simple surrogate marker for insulin resistance (IR) and is associated with the presence and severity of coronary artery disease (CAD). However, the prognostic significance of METS-IR in patients with premature CAD remains unclear. This study aims to investigate the prognostic value of METS-IR in premature CAD.

Methods: This retrospective study included 582 patients diagnosed with premature CAD between December 2012 and July 2019. The median follow-up duration was 63 months (interquartile range, 44-81 months). The primary endpoint was Major Adverse Cardiovascular Events (MACE), defined as a composite of all-cause death, non-fatal myocardial infarction (MI), repeat coronary artery revascularization, and non-fatal stroke.

Results: Patients with MACE had significantly higher METS-IR levels than those without MACE (44.88±8.11 vs. 41.68±6.87, *p*<0.001). Kaplan-Meier survival curves based on METS-IR tertiles demonstrated a statistically significant difference (log-rank test, *p*<0.001). In the fully adjusted model, the Hazard Ratio (95% CI) for MACE was 1.41 (1.16-1.72) per SD increase in METS-IR, and the P for trend based on METS-IR tertiles was 0.001 for MACE. Time-dependent Receiver Operator Characteristic (ROC) analysis of METS-IR yielded an Area Under the Curve (AUC) of 0.74 at 2 years, 0.69 at 4 years, and 0.63 at 6 years.

Conclusions: METS-IR serves as a reliable prognostic predictor of MACE in patients with premature CAD. Therefore, METS-IR may be considered a novel, cost-effective, and dependable indicator for risk stratification and early intervention in premature CAD.

## INTRODUCTION

Coronary artery disease (CAD) remains one of the leading causes of mortality and poses a significant economic burden worldwide [1, 2], and the incidence of premature CAD shows an upward trend in recent years [3–5]. Research indicated that premature CAD is a progressive disease with poor prognosis, half of patients had major adverse cardiovascular events (MACE) and one fifth populations died within a 10-year follow-up [6].

Insulin resistance (IR) is a critical aspect of both type 2 diabetes mellitus (DM) and metabolic syndrome (MetS), playing a pivotal role in the development of CAD [7–9]. Moreover, MetS is associated with higher CAD risk and has been strongly linked to premature CAD [10–13]. Besides, type 2 DM is associated with carotid artery plaques in patients with premature CAD [14]. These studies suggested that IR may be associated with prognosis of premature CAD.

Given the defect of traditional IR assessment methods (such as hyper insulinemic-euglycemic clamp and homeostatic model assessment of insulin resistance) [15], several new indicators for IR, such as METS-IR, triglyceride/high-density lipoprotein cholesterol ratio (TG/HDL-C ratio) and triglyceride and glucose index (TyG index), had been presented and showed significant predictive value of cardiovascular disease [8, 16–19]. In a cohort study of a Chinese population with 10 years of follow-up, METS-IR demonstrated greater predictive ability for both the presence and severity of CAD compared to TG/HDL-C ratio and the TyG index [20]. However, there has been limited researches exploring the association between METS-IR and MACE among patients with premature CAD. Therefore, this study aimed to explore whether METS-IR has the predictive value for MACE among patients with premature CAD.

## MATERIALS AND METHODS

### Study design and patients

This study adhered to the ethical standards outlined in the Declaration of Helsinki and obtained approval from the Ethics Review Committee of Qilu Hospital, Shandong University. The follow-up of our retrospective study was conducted via telephone, and the ethics committee granted permission for the collection of verbal consent.

**Figure 1:**
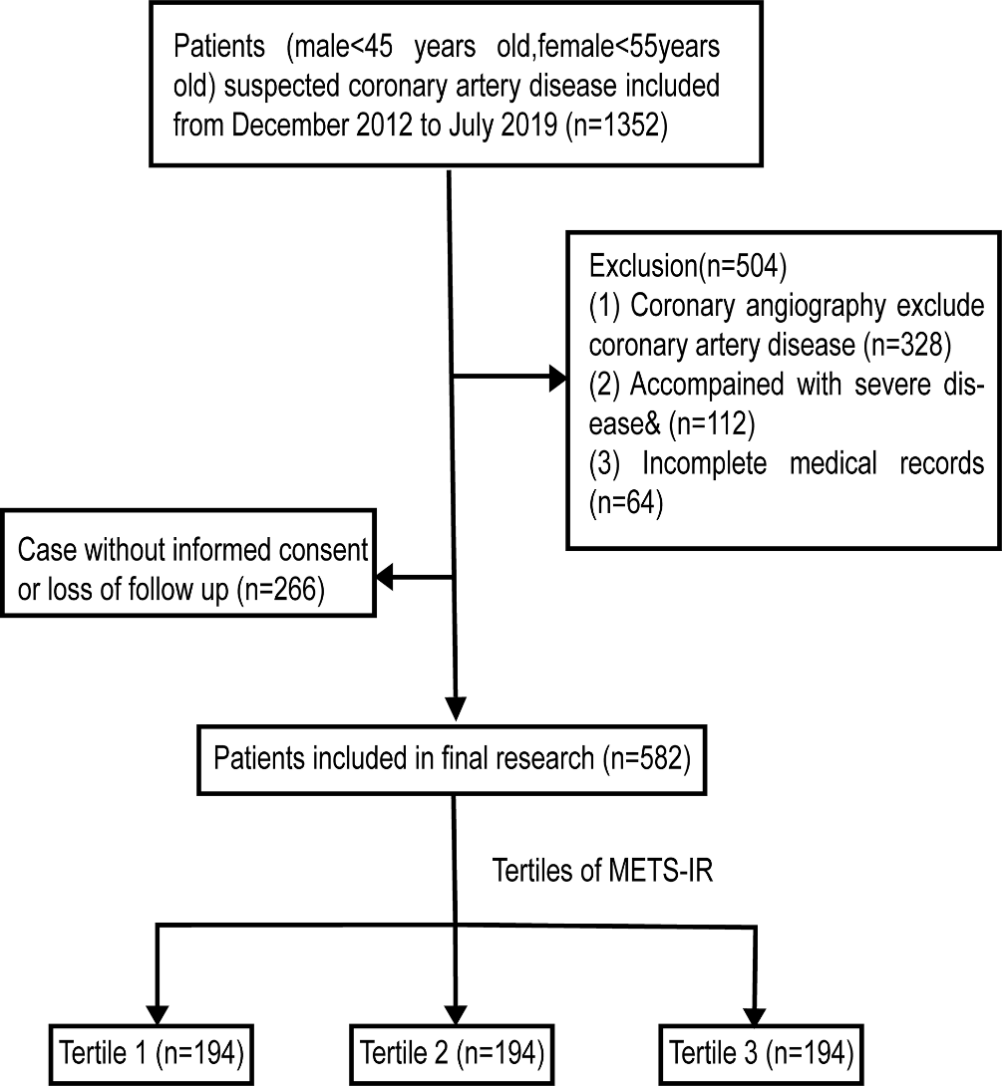
**Flow diagram of patient selection.** ^&^ Including severe cardiac value disease, decompensated heart failure, non-ischemic dilated cardiomyopathy, severe renal or hepatic disease, acute infection or inflammation, malignancy, hematologic disease or autoimmune disease.

From late September 2022, we started to collect patient data from electronic medical record system of the Qilu Hospital of Shandong University. Total of 1352 patients (female<55 years, male<45 years) who were suspected of CAD underwent coronary angiography from December 2012 to July 2019 were included. 1024 patients were diagnosed with CAD, which is determined by the existence of obstructive stenosis of >50% in any of the main coronary arteries, consisting of the left main coronary artery (LM), right coronary artery (RCA), left anterior descending artery (LAD) and left circumflex coronary artery (LCX) [21]. Patients with incomplete medical records (n=64) or severe disease [including severe cardiac value disease, decompensated heart failure, non-ischemic dilated cardiomyopathy, severe hepatic disease or renal (liver function parameters>3 × upper normal value or serum creatinine>1.4 mg/dL), acute infection or inflammation, autoimmune disease or hematologic disease and malignancy, n=112] were removed from this study. 848 patients were admitted in the study and followed up from October 2022 to November 2022. 582 patients (68.6%) completed the phone follow-up after providing verbal consent (Figure 1).

### Definitions and the process of data collection

Practicing clinicians gathered clinical data from medical records. The data included general conditions [age, gender, body mass index (BMI), left ventricular ejection fraction (LVEF), multivessel disease, admission for myocardial infarction(MI) and Gensini score (GS)], cardiovascular risk factors [family history of CAD (FH-CAD), current smoking, hypertension and DM], laboratory tests [total cholesterol (TC), fasting plasma glucose (FPG), low-density lipoprotein cholesterol (LDL-C), triglyceride (TG), high-density lipoprotein cholesterol (HDL-C), uric acid (UA) and serum creatinine (SCr)] and discharge medications [statins, antiplatelet drugs, angiotensin-converting enzyme inhibitors (ACEI), angiotensin receptor blockers (ARB), beta-blockers, oral hypoglycemic drugs and insulins]. To calculate BMI, the weight in kilograms is divided by the square of the height in meters. MI was diagnosed by 12-lead electrocardiography, analysis of serum myocardial enzymes and echocardiograph [22]. The severity of CAD was assessed by the results of coronary angiography and was expressed as GS [23]. Multivessel disease was defined as more than 1 major coronary artery existing ≥50% diameter stenosis. FH-CAD was defined as having a history of CAD in a first-degree relative who is younger than 65 years of age for women or younger than 55 years of age for men. The definition of DM is based on specific glucose levels: a FPG level of 7.0 mmol/L or higher, a random blood glucose level of 11.1 mmol/L or higher and a 2 h plasma glucose after oral glucose tolerance test (OGTT) of 11.1 mmol/L or higher [24] or use of oral hypoglycemic agents or insulin. The definition of hypertension was: systolic blood pressure (SBP) ≥ 140 mmHg and/or diastolic blood pressure (DBP) ≥ 90 mmHg or use of antihypertensive medication. According to the Modification of Diet in Renal Disease (MDRD) equations, the estimated glomerular filtration rate (eGFR) was determined through the application of the following formula: 175 × SCr(mg/dL)^−1.234^ × age (year)^−0.179^ × 0.79 (if female) [25]. The calculation of METS-IR was determined as follows: ln [(2 × FPG (mg/dL)) + fasting TG (mg/dL)] × BMI (kg/m^2^**)** ÷ Ln [HDL-C (mg/dL)] [26].

### Endpoints

In the study, the primary endpoint was to determine the rate of MACE in patients with premature CAD. MACE was defined as the composite endpoint encompassing of all-cause, repeat coronary artery revascularization [coronary artery bypass graft surgery (CABG) or percutaneous coronary intervention (PCI)], non-fatal MI, and non-fatal stroke. The secondary endpoints involved all-cause death, non-fatal stroke, non-fatal MI and repeat coronary artery revascularization.

### Statistical analysis

Statistical analysis was completed through SPSS version 26.0 and R software version 4.2.1. During the follow-up period, participants were categorized based on the occurrence of MACE. The METS-IR values were divided into three groups by tertiles as follows: Tertile1(METS-IR <39.18), Tertile2(39.18 ≤ METS-IR < 45.01) and Tertile3(METS-IR≥45.01). Continuous variables were presented in terms of their mean ± standard deviation (SD) or their median values along with the 25th and 75th percentiles. For normally distributed continuous variables, the independent samples t-test or ANOVA test was employed. Conversely, for non-normally distributed continuous variables, the Mann-Whitney U-test or Kruskal-Wallis H-test was used. Categorical variables were presented as counts and percentages, and comparisons were made using the chi-square test or Fisher's exact test. Spearman or Pearson correlation analysis was used to explore the correlations between METS-IR and cardiovascular indicators, excluding those indicators used in the calculation of METS-IR, which reflected the degree of coronary stenosis, cardiac function, renal function, and metabolism. To evaluate the cumulative occurrence of MACE and secondary endpoints, Kaplan–Meier curves were utilized. The log-rank test was used to determine whether the distribution of cumulative MACE and secondary endpoints incidence differed among the groups. Variables were analyzed by univariate Cox regression analysis. Multivariate Cox proportional hazards regression was accomplished to determine whether the METS-IR can independently predict the occurrence of MACE. To assess the prediction of METS-IR with multiple confounders in occurrence of MACE, we built three Cox regression models: model 1 was adjusted for gender and age; model 2 was adjusted for variables with *p*<0.05 in univariate Cox analysis; and model 3 was adjusted for entire variables which involved age, LVEF, gender, admission for MI, GS, multivessel disease, FH-CAD, current smoking, hypertension, DM, TC, LDL-C, UA, eGFR, ACEI/ARB, antiplatelet drugs, statins, oral hypoglycemic drugs and insulins. The METS-IR was incorporated into the models both as continuous variables and categorical variables (the tertile of METS-IR), respectively. The standardization of METS-IR was used to determine the prediction of METS-IR per SD increase. To avoid the result deviation caused by multicollinearity, we calculated the variance inflation factor (VIF) of the variables included in the models. According to VIF<10, there is no multicollinearity in models. We further observed the relationship between METS-IR and outcome events using restricted cubic spline (RCS). Subgroups analysis which was based on gender, current smoking, FH-CAD, DM, and hypertension was completed and the *p* for interaction was also calculated by multiplication interaction term. The area under the curve (AUC) in time-dependent receiver operating characteristic (ROC) curves were used to explore the predictive value of METS-IR in different times. To determine whether an increased METS-IR had additional predictive value in MACE, repeat coronary artery revascularization and non-fatal MI, model 3 without and with METS-IR were compared in C-Statistic, integrated discrimination improvement (IDI) and continuous net reclassification improvement (NRI). Furthermore, model 3 without and with METS-IR were compared in Bayesian Information Criterion (BIC) and Akaike Information Criterion (AIC) by likelihood ratio test. Furthermore, we excluded endpoints occurring in the first six months of follow-up and performed sensitivity analysis. Statistical significance was determined when the p-value was less than 0.05.

### Data sharing statement

The datasets used and/or analyzed in the study are available from the corresponding author upon reasonable request.

## RESULTS

### Baseline characteristics

The mean age of the patients was 44.43±6.33 years and 352 (60.5%) patients were male. According to the occurrence of MACE, the baseline characteristics were presented in Table 1. Patients with MACE were those tended to be smokers (*p*=0.022), or to have FH-CAD(*p*=0.037), DM(*p*<0.001), hypertension(*p*=0.024) or multivessel disease(*p*<0.001). BMI(*p*=0.010), GS(*p*=0.023), FPG(*p*<0.001), TG(*p*=0.002), HDL-C(*p*=0.022) and use of oral hypoglycemic drugs (*p*=0.013) and insulins(*p*<0.001) were also significantly different between the two groups. Furthermore, patients who experienced MACE had a significantly higher level of METS-IR compared to those who did not experience MACE (44.88±8.11 vs 41.68±6.87, p<0.001). As presented in Table 2, patients were divided into three groups based on the tertiles of METS-IR (Tertile1: n=194, METS-IR < 39.18; Tertile2: n=194, 39.18 ≤ METS-IR < 45.01; Tertile3: n=194, METS-IR≥45.01). Significant differences were found among three groups in terms of age, gender, BMI, GS, current smoking, FH-CAD, DM, FPG, TC, TG, HDL-C, UA, ACEI/ARB, oral hypoglycemic drugs, insulins, MACE, non-fatal MI, repeat coronary artery revascularization (Table 2). In addition, we compared the baseline data of the population finally included in the study and those who lost follow-up and found no significant difference (Supplementary Material: Supplementary Table 1).

**Table 1:** Baseline characteristics of the study population according to the occurrence of MACE.

**Variables**	**Total (n =582)**	**Without event (n =464)**	**With event (n=118)**	***p*-value**
**General conditions**				
Age (years)	44.43±6.33	44.57±6.16	43.92±6.97	0.355
Male, n (%)	352(60.5)	286(61.6)	66(55.9)	0.258
BMI (kg/m^2^)	26.89±3.35	26.68±3.15	27.71±3.94	**0.010**
LVEF (%)	60.00(56.00-65.00)	60.00(56.00-65.00)	60.00(55.00-64.00)	0.224
Admission for MI, n (%)	161(27.7)	126(27.2)	35(29.7)	0.587
GS	36.75(21.00-61.00)	34.00(20.00-58.75)	45.00(24.75-67.00)	**0.023**
Multivessel disease, n (%)	261(44.8)	187(40.3)	74(62.7)	**<0.001**
**Risk factors, n (%)**				
Current Smoking	172(29.6)	127(27.4)	45(29.6)	**0.022**
FH-CAD	167(28.7)	124(26.7)	43(36.4)	**0.037**
DM	124(21.3)	82(17.7)	42(25.6)	**<0.001**
Hypertension	321(55.2)	245(52.8)	76(64.4)	**0.024**
**Laboratory test**				
FPG (mg/dL)	89.02(81.09-108.03)	88.30(80.23-102.35)	99.11(83.57-134.77)	**<0.001**
TC (mg/dL)	154.45(129.12-185.28)	152.71(128.83-183.92)	158.51(132.70-190.01)	0.252
TG (mg/dL)	128.39(97.44-180.05)	124.85(94.96-176.87)	146.54(112.23-188.60)	**0.002**
LDL-C (mg/dL)	95.10(74.13-118.40)	93.36(73.07-116.75)	99.55(75.00-124.10)	0.186
HDL-C (mg/dL)	41.37(35.57-49.10)	42.14(35.95-50.26)	39.82(34.99-44.94)	**0.022**
eGFR (mL/min/1.73m^2^)	114.52(100.54-130.45)	114.03(99.71-130.04)	118.34(105.19-131.42)	0.130
UA (μmol/L)	318.50(257.00-371.25)	320.50(258.00-371.00)	307.00(253.75-382.50)	0.650
**Discharge medications, n (%)**				
Antiplatelet drugs	551(94.7)	438(94.4)	113(95.8)	0.555
Stains	555(95.4)	445(95.9)	110(93.2)	0.216
Beta-blockers	369(63.4)	297(64.0)	72(61.0)	0.547
ACEI/ARB	265(45.5)	207(44.6)	58(49.2)	0.377
Oral hypoglycemic drugs	66(11.3)	45(9.7)	21(17.8)	**0.013**
Insulins	25((4.3)	13(2.8)	12(10.2)	**<0.001**
**Index**				
METS-IR	42.33±7.25	41.68±6.87	44.88±8.11	**<0.001**

Data were given as mean ± SD, median with interquartile range or n (%).

MACE, major adverse cardiovascular events; BMI, body mass index; LVEF, left ventricle ejection fraction; MI, myocardial infarction; GS, Gensini score; FH-CAD, family history of coronary artery disease; DM, diabetes mellitus; FPG, fasting plasma glucose; TC, total cholesterol; TG, triglyceride; LDL-C, low-density lipoprotein-cholesterol; HDL-C, high-density lipoprotein-cholesterol; eGFR, estimated glomerular filtration rate; UA, uric acid; ACEI, angiotensin-converting enzyme inhibitors; ARB, angiotensin receptor blockers; METS-IR, metabolic score for insulin resistance.

*p*-values in bold are<0.05.

**Table 2:** Baseline characteristics of the study population according to the tertiles of the METS-IR

**Variables**	**Tertile1 (n=194)**	**Tertile2 (n=194)**	**Tertile3 (n=194)**	***p*-values**
**General conditions**				
Age (years)	45.93±6.16	45.40±5.84	41.97±6.27	**<0.001**
Male, n (%)	95(49.0)	120(61.9)	137(70.6)	**<0.001**
BMI (kg/m2)	23.86±2.23	26.92±1.79	29.89±2.69	**<0.001**
LVEF (%)	61.00(58.00-66.25)	60.00(55.00-65.00)	60.00(56.00-65.00)	0.142
Admission for MI, n (%)	44(22.7)	56(28.9)	61(31.4)	0.140
GS	27.00(18.00-50.00)	40.00(22.75-62.00)	44.00(23.00-73.25)	**<0.001**
Multivessel disease, n (%)	84(43.3)	90(46.4)	87(44.8)	0.829
**Risk factors, n (%)**				
Current Smoking	37(19.1)	58(29.9)	77(39.7)	**<0.001**
FH-CAD	42(21.6)	51(26.3)	74(38.1)	**0.001**
DM	27(13.9)	37(19.1)	60(30.9)	**<0.001**
Hypertension	100(51.1)	105(54.1)	116(59.8)	0.248
**Laboratory test**				
FPG (mg/dL)	85.23(78.03-94.15)	88.84(80.71-106.36)	95.69(84.38-129.46)	**<0.001**
TC (mg/dL)	161.99(134.92-194.65)	150.97(128.25-179.00)	151.35(120.04-179.87)	**0.004**
TG (mg/dL)	110.24(85.00-133.70)	128.39(95.67-170.23)	169.62(124.63-231.99)	**<0.001**
LDL-C (mg/dL)	98.39(75.68-122.17)	92.78(75.00-118.01)	93.75(70.65-116.97)	0.470
HDL-C (mg/dL)	49.68(43.20-55.67)	40.59(36.63-46.78)	35.95(31.31-40.21)	**<0.001**
eGFR (mL/min/1.73m2)	114.89(102.25-130.63)	114.22(101.18-129.55)	114.77(97.55-132.01)	0.983
UA (μmol/L)	294.00(238.75-356.00)	307.00(250.75-362.25)	349.50(293.50-397.00)	**<0.001**
**Discharge medications, n (%)**				
Antiplatelet drugs	182(98.8)	184(94.3)	185(95.4)	0.788
Stains	185(95.4)	187(96.4)	183(94.3)	0.627
Beta-blockers	112(57.7)	131(67.5)	126(64.9)	0.116
ACEI/ARB	70(36.1)	91(46.9)	104(53.6)	**0.002**
Oral hypoglycemic drugs	13(6.7)	21(10.8)	32(16.5)	**0.009**
Insulins	4(2.1)	6(3.1)	15(7.7)	**0.014**
**Outcomes, n (%)**				
MACE	24(12.4)	36(18.6)	58(29.9)	**<0.001**
All-cause death	2(1.0)	2(1.0)	4(2.1)	0.602
Non-fatal MI	6(3.1)	13(6.7)	19(9.8)	**0.028**
Repeat coronary artery revascularization	14(7.2)	20(10.3)	35(18.0)	**0.003**
Non-fatal stroke	2(1.0)	1(0.5)	1(0.5)	0.777

Data were given as mean ± SD, median with interquartile range or n (%).

METS-IR, metabolic score for insulin resistance; BMI, body mass index; LVEF, left ventricle ejection fraction; MI, myocardial infarction; GS, Gensini score; FH-CAD, family history of coronary artery disease; DM, diabetes mellitus; FPG, fasting plasma glucose; TC, total cholesterol; TG, triglyceride; LDL-C, low-density lipoprotein-cholesterol; HDL-C, high-density lipoprotein-cholesterol; eGFR, estimated glomerular filtration rate; UA, uric acid; ACEI, angiotensin-converting enzyme inhibitors; ARB, angiotensin receptor blockers; MACE, major adverse cardiovascular events.

*p-*values in bold are<0.05.

### Correlations between METS-IR and traditional cardiovascular indicators

The correlation between METS-IR and traditional cardiovascular indicators was tested using Pearson or Spearman correlation analysis. Table 3 showed that METS-IR was positively associated with GS and UA, and negatively associated with age (*p*<0.05). There was no significant correlation between METS-IR and LVEF, LDL-C, and eGFR.

**Table 3:** Correlations between the METS-IR and cardiovascular risk factors.

**Variables**	**Correlation coefficient**	***p*-value**
Age, years	-0.300 ^P^	**<0.001**
LVEF, %	-0.057 ^S^	0.169
GS	0.188 ^S^	**<0.001**
LDL-C, mmol/L	-0.025 ^S^	0.552
eGFR, mL/min/1.73m^2^	-0.041 ^S^	0.320
UA, μmol/L	0.274 ^S^	**<0.001**

METS-IR, metabolic score for insulin resistance; LVEF, left ventricle ejection fraction; GS, Gensini score; LDL-C, low-density lipoprotein-cholesterol; eGFR, estimated glomerular filtration rate; UA, uric acid.

*p*-values in bold are<0.05.

^P^Pearson correlation analysis.

^S^Spearman correlation analysis.

### METS-IR and MACE

**Figure 2:**
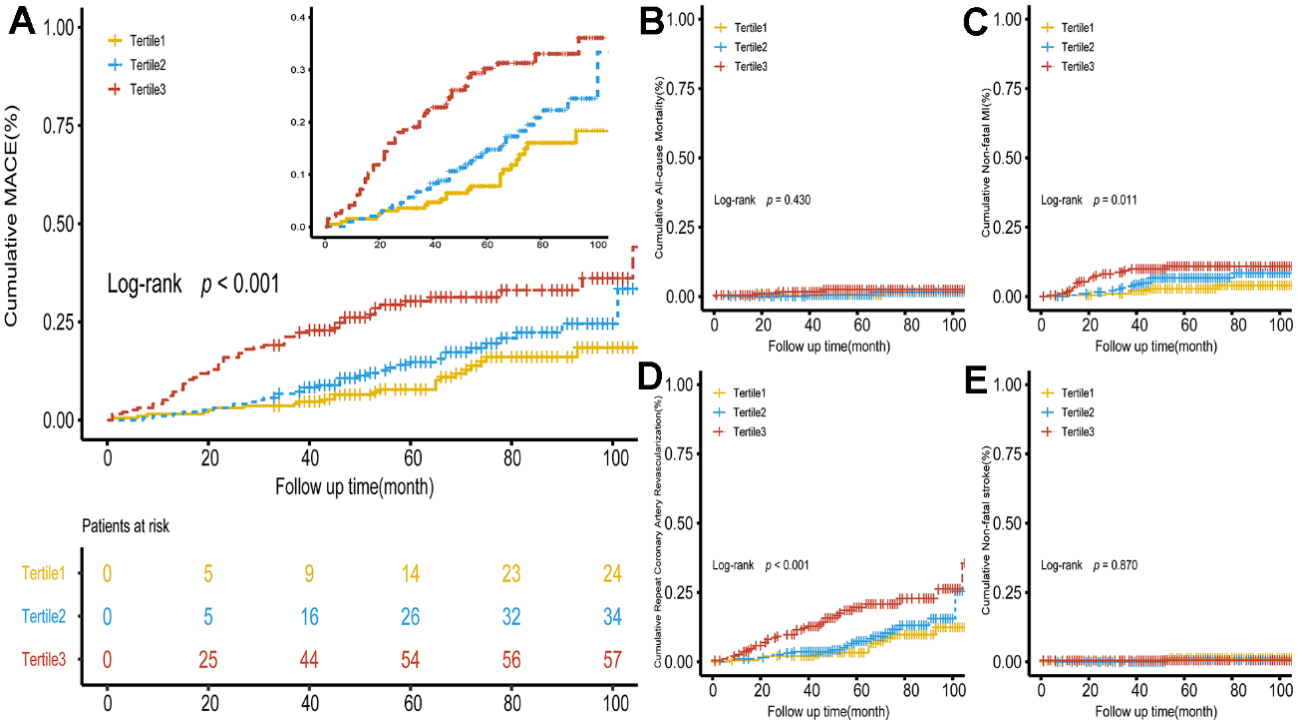
**Kaplan–Meier survival curve for MACE and secondary endpoints across the METS-IR tertiles.** (**A**) Significance was found between MACE and METS-IR tertiles. (**B**–**E**) Cumulative incidence curves for all-cause death, non-fatal infarction, repeat coronary artery revascularization and non-fatal shock, respectively. METS-IR tertiles were significantly associated with repeat coronary artery revascularization and non-fatal MI. There was no significance in all-cause death and non-fatal stroke. METS-IR, metabolic score for insulin resistance; MACE, major adverse cardiovascular events.

During the 63 months (interquartile range, 44-81 months) follow-up, 118 (20.27%) MACEs were recorded, including 8 (1.37%) all-cause death, 38 (6.53%) non-fatal MI, 69 (11.86%) repeat coronary artery revascularization and 4 (0.69%) non-fatal strokes. Kaplan–Meier survival plots (Figure 2) demonstrated that the higher METS-IR group had a significantly elevated cumulative incidence of MACE, non-fatal MI and repeat coronary artery revascularization (MACE: log-rank test, *p* < 0.001; non-fatal MI: log-rank test, *p* = 0.011; repeat coronary artery revascularization; log-rank test, *p* < 0.001). As shown in Table 4, BMI, multivessel disease, current smoking, FH-CAD, DM, hypertension, FPG, TG and METS-IR were related with the risk of MACE, while HDL-C was a protective factor for MACE, use of statins can reduce risk of MACE. In addition, patients who need to take oral hypoglycemic drugs or insulins had a higher risk of MACE. The adjusted HR (95% CI) for risk of MACE with per SD increase in METS-IR was 1.59 (1.32–1.87) (Table 4). Whether regarded as continuous or categorical variable, the METS-IR kept significant after adjusting for confounders. In model 3, per SD increase in METS-IR, a 41.1% (HR=1.41; 95%CI 1.16-1.72) increase was found in the risk of developing MACE. Compared with patients in the lowest tertile, the adjusted HR (model 3) for MACE was 1.58(0.93-2.70) and 2.48(1.45-4.26) in the middle and highest tertile. Significantly increased risk of MACE from tertile1 to tertile3 of METS-IR (*p* for trend<0.001) was found (Table 5). The associations between METS-IR and non-fatal MI and repeat coronary artery revascularization were also examined (Table 6). RCS was used to further explore the association between METS-IR and MACE, repeat coronary artery revascularization and non-fatal MI (Supplementary Material: Supplementary Figures 1–3).

**Table 4:** Univariate Cox regression analysis for MACE.

**Variables**	**HR**	**95%CI**	***p*-value**
Age	0.99	0.96-1.01	0.299
Male	0.78	0.54-1.13	0.189
BMI	1.09	1.04-1.15	**0.001**
LVEF	0.46	0.08-2.63	0.381
Admission for MI	1.04	0.70-1.55	0.842
GS	1.01	1.00-1.01	0.083
Multivessel disease	1.79	1.23-2.60	**0.002**
Current Smoking	1.60	1.11-2.32	**0.013**
FH-CAD	1.59	1.09-2.31	**0.016**
DM	2.33	1.60-3.40	**<0.001**
Hypertension	1.57	1.08-2.29	**0.019**
FPG	1.01	1.01-1.01	**<0.001**
TC	1.00	1.00-1.01	0.085
TG	1.00	1.00-1.00	**0.001**
LDL-C	1.00	1.00-1.01	0.082
HDL-C	0.97	0.95-0.99	**0.005**
eGFR	1.01	1.00-1.01	0.117
UA	1.00	1.00-1.00	0.630
Antiplatelet drugs	0.97	0.36-2.39	0.952
Stains	0.46	0.22-0.95	**0.037**
Beta-blockers	0.90	0.63-1.31	0.595
ACEI/ARB	1.05	0.73-1.50	0.807
Oral hypoglycemic drugs	1.91	1.19-3.06	**0.007**
Insulins	2.81	1.55-5.12	**0.001**
METS-IR	1.01	1.04-1.09	**<0.001**
Standardization of METS-IR	1.59	1.32-1.87	**<0.001**

MACE, major adverse cardiovascular events; HR, Hazard ratio; CI, Confidence interval; BMI, body mass index; LVEF, left ventricle ejection fraction; MI, myocardial infarction; GS, Gensini score; FH-CAD, family history of coronary artery disease; DM, diabetes mellitus; FPG, fasting plasma glucose; TC, total cholesterol; TG, triglyceride; LDL-C, low-density lipoprotein-cholesterol; HDL-C, high-density lipoprotein-cholesterol; eGFR, estimated glomerular filtration rate; UA, uric acid; ACEI, angiotensin-converting enzyme inhibitors; ARB, angiotensin receptor blockers; METS-IR, metabolic score for insulin resistance.

*p*-values in bold are<0.05.

**Table 5:** Multivariate Cox regression analysis for MACE.

**METS-IR**	**HR (95% CI)**		
	**Model 1**	**Model 2**	**Model 3**
Per 1 Unit increase	1.07(1.04-1.09) **	1.05(1.02-1.07) **	1.05(1.02-1.08) *
Per 1 SD increase	1.61(1.35-1.92) **	1.39(1.16-1.67) **	1.41(1.16-1.72) *
Tertile1	1(Reference)	1(Reference)	1(Reference)
Tertile2	1.70(1.01-2.87) *	1.42(0.84-2.39)	1.58(0.93-2.70)
Tertile3	3.26(2.00-5.33) **	2.37(1.40-3.83) *	2.48(1.45-4.26) *
*p* for trend	**<0.001**	**0.001**	**0.001**

Model 1: adjusted for age and gender.

Model 2: adjusted for variables with *p*-value<0.05 in univariate Cox regression analysis for MACE, including, multivessel disease, current Smoking, FH-CAD, DM, Hypertension, stains, Oral hypoglycemic drugs, Insulins.

Model 3: adjusted for age, gender, LVEF, admission for MI, multivessel disease, GS, current smoking, FH-CAD, DM, hypertension, TC, LDL-C, eGFR, UA, antiplatelet drugs, statins, betablockers, ACEI/ARB, Oral hypoglycemic drugs, Insulins.

MACE, major adverse cardiovascular events; METS-IR, metabolic score for insulin resistance; HR, hazard ratio; CI, confidence interval; SD, standard deviation.

*p*-values in bold are <0.05.

^*^p<0.05.

^**^p<0.001.

**Table 6:** Multivariate Cox regression analyses for coronary artery revascularization and non-fatal MI.

**METS-IR**	**HR (95% CI)**		
	**Model 1**	**Model 2**	**Model 3**
**Repeat coronary artery revascularization**			
Per 1 Unit increase	1.06(1.03-1.09) *	1.04(1.01-1.08) *	1.04(1.00-1.08) *
Per 1 SD increase	1.52(1.20-1.92) *	1.37(1.07-1.75) *	1.33(1.02-1.72) *
Tertile1	1(Reference)	1(Reference)	1(Reference)
Tertile2	1.60(0.81-3.20)	1.30(0.65-2.59)	1.40(0.69-2.84)
Tertile3	3.17(1.67-6.01) **	2.43(1.26-4.68) *	2.37(1.18-4.79) *
*p* for trend	**<0.001**	**0.005**	**0.014**
**Non-fatal MI**			
Per 1 Unit increase	1.08(1.04-1.13) **	1.06(1.02-1.11) *	1.08(1.03-1.13) *
Per 1 SD increase	1.76(1.31-2.36) **	1.55(1.15-2.10) *	1.69(1.20-2.38) *
Tertile1	1(Reference)	1(Reference)	1(Reference)
Tertile2	2.48(0.94-6.55)	2.00(0.76-5.31)	2.48(0.90-6.87)
Tertile3	4.32(1.69-11.07) *	3.10(1.19-8.09) *	3.89(1.39-10.88) *
*p* for trend	**0.002**	**0.017**	**0.009**

Model 1: adjusted for age and gender.

Model 2: adjusted for variables with p-value<0.05 in univariate Cox regression analysis for MACE, including, multivessel disease, current Smoking, FH-CAD, DM, Hypertension, stains, Oral hypoglycemic drugs, Insulins.

Model 3: adjusted for age, gender, LVEF, admission for MI, multivessel disease, GS, current smoking, FH-CAD, DM, hypertension, TC, LDL-C, eGFR, UA, antiplatelet drugs, statins, betablockers, ACEI/ARB, Oral hypoglycemic drugs, Insulins.

Non-fatal MI, non-fatal myocardial infraction; METS-IR, metabolic score for insulin resistance; HR, hazard ratio; CI, confidence interval; SD, standard deviation.

*p*-values in bold are <0.05.

*p<0.05.

**p<0.001.

### Subgroup analysis

**Figure 3:**
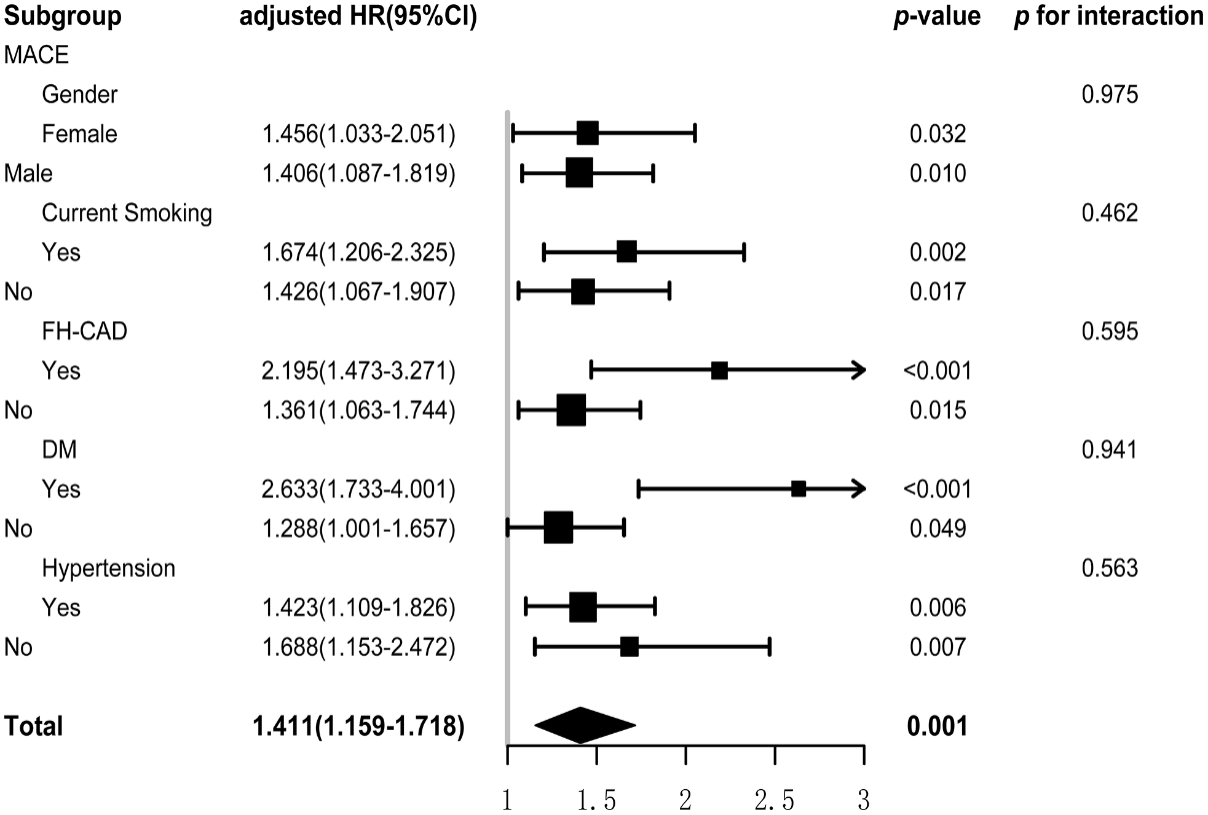
**Subgroup and interaction analysis between METS-IR (Per SD) and MACE in various subgroup.** METS-IR, metabolic score for insulin resistance; MACE, major adverse cardiovascular events.

Subgroup analysis, based on the gender, current smoking, FH-CAD, DM, and hypertension, was used to examine the association between METS-IR and MACE. There was non-significant interaction found in this subgroup analysis. Significance was observed in every subgroup. The evidence suggested that METS-IR was a significant risk factor for incidence of MACE in general premature CAD patients (Figure 3).

### Assessment of the prognostic capability of METS-IR for MACE

**Figure 4:**
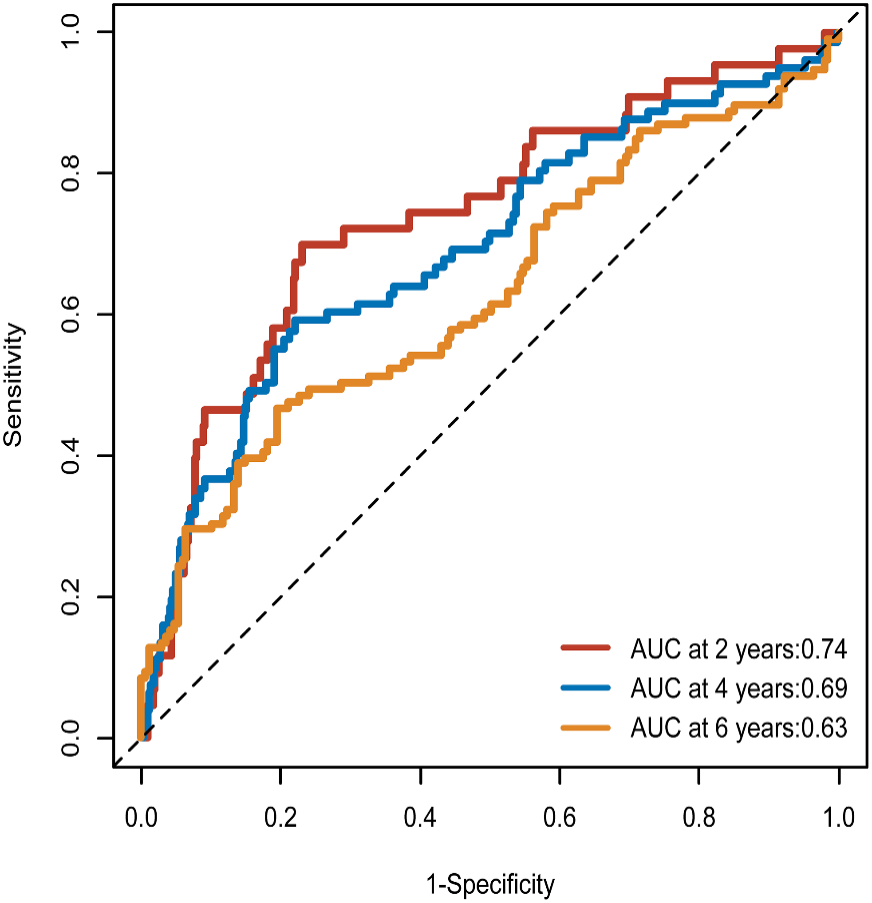
**Time-dependent ROC curves of the METS-IR for the prediction of MACE.** ROC curve, receiver operating characteristic curve; METS-IR, metabolic score for insulin resistance; MACE, major adverse cardiovascular events; AUC, area under the curves.

The AUC was 0.74 at 2 years, 0.69 at 4 years, and 0.63 at 6 years (Figure 4). The incremental predictive value of METS-IR for MACE, repeat coronary artery revascularization and non-fatal MI were presented in Table 7. According to C-Statistic, risk prediction for MACE was significantly improved after adding METS-IR to existing risk prediction model (C-Statistic increased from 0.71 to 0.72, *p*<0.001). Additionally, the improvement in risk prediction of METS-IR for MACE was also reflected in NRI and IDI [Continuous NRI (95%CI) = 0.19 (-0.01-0.39), *p*=0.061; IDI (95%CI) = 0.02(0.00-0.03), *p*=0.007]. Significant improvement was also discovered in risk prediction for non-fatal MI and repeat coronary artery revascularization. Compared with model 3 without METS-IR, the AIC and BIC were significantly improved in predicting MACE and non-fatal MI and significant improvement of AIC was only observed in predicting repeat coronary artery revascularization (Table 8).

**Table 7:** The incremental predictive value of the METS-IR index.

	**C-index (95%CI)**	***p*-value**	**Continuous NRI (95%CI)**	***p*-value**	**IDI (95%CI)**	***p*-value**
**MACE**						
Model 3 without METS-IR	0.71(0.66-0.76)		Reference		Reference	
Model 3 with METS-IR	0.72(0.67-0.77)	**<0.001**	0.19(-0.01-0.39)	0.061	0.02(0.00-0.03)	**0.007**
**Repeat coronary artery revascularization**						
Model 3 without METS-IR	0.75(0.68-0.81)		Reference		Reference	
Model 3 with METS-IR	0.76(0.70-0.82)	**0.038**	0.09(-0.11-0.30)	0.363	0.01(0.00-0.01)	**0.009**
**Non-fatal MI**						
Model 3 without METS-IR	0.71(0.62-0.80)		Reference		Reference	
Model 3 with METS-IR	0.74(0.64-0.83)	**0.003**	0.18(-0.02-0.38)	0.079	0.01(0.00-0.02)	**0.010**

METS-IR, metabolic score for insulin resistance; MACE, major adverse cardiovascular events; Non-fatal MI, non-fatal myocardial infarction; NRI, net reclassification improvement; IDI, integrated discrimination improvement.

The time point for the calculation of the C-index, continuous NRI and IDI is the end of follow-up, which is approximately 10 years.

*p*-values in bold are <0.05.

**Table 8:** Assessment of the goodness-of -fit of models.

	**Model 3 without METS-IR**	**Model 3 with METS-IR**	***p*-value**
**MACE**			
AIC	1387.06	1377.58	**<0.001**
BIC	1442.48	1435.76	**<0.001**
df	Reference	1	
**Repeat coronary artery revascularization**			
AIC	797.26	794.98	**0.033**
BIC	841.95	841.90	0.754
df	Reference	1	
**Non-fatal MI**			
AIC	484.95	478.28	**<0.001**
BIC	517.70	512.67	**0.002**
df	Reference	1	

METS-IR, metabolic score for insulin resistance; MACE, major adverse cardiovascular events; Non-fatal MI, non-fatal myocardial infarction; AIC, Akaike information criterion; BIC, Bayesian information criterion; df, degree of freedom.

The time point for the calculation of the AIC and BIC is the end of follow-up, which is approximately 10 years.

*p*-values in bold are < 0.05.

### Sensitivity analysis

To control the effect of PCI surgical quality and anticoagulant administration in occurrence of study endpoints, we excluded endpoints occurring in first six months of follow-up and performed sensitivity analysis. The results of the sensitivity analysis were consistent with the results of the initial analysis (Supplementary Material: Supplementary Table 2).

## DISCUSSION

To the best of our knowledge, this study is the inaugural one to uncover the association between METS-IR and premature CAD. Our study suggested that (1) METS-IR is an independent risk factor for developing MACE in patients with premature CAD. (2) Adding METS-IR to existing risk prediction models improves the prognostic power of MACE in patients with premature CAD. In conclusion, our study revealed the prognostic value of MACE in patients with premature CAD.

Although CAD mainly happened in elder, the incidence of CAD in younger shows an upward trend and is frequently linked to unfavorable outcomes [3–5]. Previous researches showed IR independently predicts risk of cardiovascular diseases [7–9]. Although the Hyperinsulinemic-euglycemic clamp technique (HEC) is considered the gold standard to assess IR, it is costly and time-consuming [15]. Despite HOMA-IR is a simple indicator for IR and exists a reasonable linear correlation with HEC, HOMA-IR may not yield accurate results in individuals who have severely impaired or absent β-cell function [15]. As a simple surrogate for IR, METS-IR has been proven to be a predictive indicator for cardiovascular disease [19, 27]. Nevertheless, the prognostic value of METS-IR for MACE in patients of premature CAD was poorly known.

Previous research has demonstrated a positive association between METS-IR and an elevated risk of cardiovascular disease [27]. In this study, we examined the relationship between METS-IR and other cardiovascular indicators in patients with premature CAD and assessed the severity of premature CAD using GS. Our findings determined a positive association between METS-IR and GS, indicating that METS-IR may serve as a surrogate for assessing coronary lesion severity in young individuals. Previous research has indicated a positive correlation between METS-IR and age in CAD patients [27]. Nevertheless, negative correlation between METS-IR and age was determined in our current study. In response to this result, we propose a plausible hypothesis that more powerful insulin compensation ability in younger patients may be a possible cause. With the increase of IR, the body makes compensation by increasing insulin secretion. However, β-Cell function is compromised in aging [28]. Younger patients are better able to compensate for insulin needed to maintain normal metabolic activity. With the increasing IR, endocrine homeostasis may be imbalanced and lead to abnormal glucose and lipid metabolism, which eventually causes CAD. However, the negative correlation and the potential mechanisms between METS-IR and age among patients with premature CAD needs to be verified by more studies.

Multivariate Cox regression analysis demonstrated that the MTES-IR was an independent risk factor for occurrence of MACE in patients with premature CAD. Additionally, subgroups analysis determined that the association between METS-IR and MACE among premature CAD patients may be independent of smoking, FH-CAD, DM and hypertension. Previous study has determined that a non-insulin-dependent IR indicator is independent prognostic indicator for MACE in premature CAD patients [29]. Smoking, FH-CAD and DM as traditional cardiovascular risk factors [30–32], have synergistic effect with METS-IR in promoting MACE occurrence. Additionally, the sensitivity analysis results did not yield significant changes. Based on the aforementioned findings, it was determined that METS-IR serves as a reliable predictor of MACE.

Several limitations of this study should be considered. Firstly, our study had limitations due to its retrospective nature, small sample size, and being conducted at a single center. This led to potential biases and limited our ability to explore the association between METS-IR and MACE, especially among patients with all-cause death or non-fatal stroke, as the number of such cases was relatively small. Secondly, potential bias stemming from measurement error was introduced as laboratory parameters were only assessed once during the study. Thirdly, despite METS-IR serving as an indicator of insulin resistance, HOMA-IR is the gold standard for reflecting insulin resistance, but our retrospective study design resulted in us not being able to obtain HOMA-IR. Therefore, we could not demonstrate that the predictive value of METS-IR was not weaker than HOMA-IR. Prospective studies may further corroborate our findings.

## CONCLUSIONS

The findings indicate a positive association between the METS-IR index and the incidence of MACE among patients with premature CAD. Therefore, METS-IR can serve as a valuable tool for identifying patients at an elevated risk of developing MACE, thus facilitating risk stratification in this patient population.

## Supplementary Material

Supplementary Figures

Supplementary Tables
