# Supplementary material for: Metabolic score for insulin resistance predicts major adverse cardiovascular event in premature coronary artery disease
Source: Aging (Albany NY). 2024 Apr 1;16(7):6364–83. doi: 10.18632/aging.205710 (PMC11042949; doi:10.18632/aging.205710)
Supplement: Supplementary Figures [file aging-16-205710-s001.pdf]

SUPPLEMENTARY FIGURES

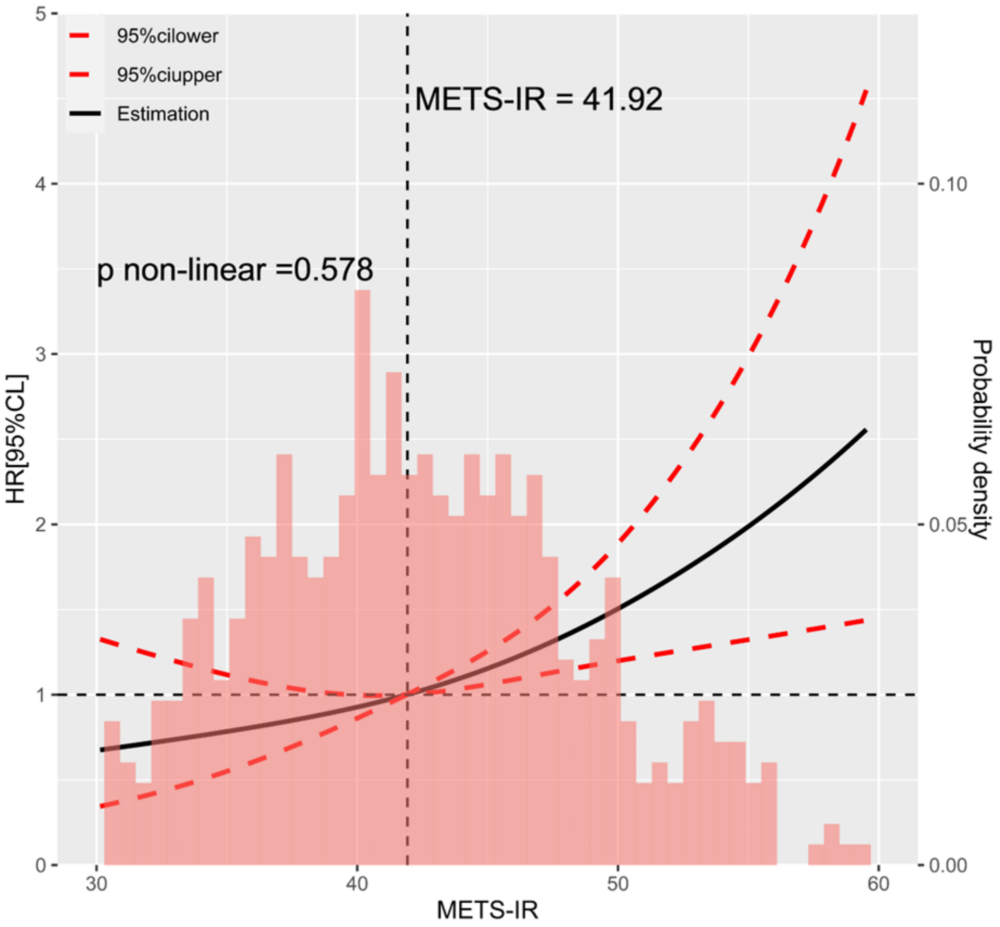

Supplementary Figure 1. The association between METS-IR and MACE. Adjusted for age, gender, LVEF, admission for MI, multivessel disease, GS, current smoking, FH-CAD, DM, hypertension, TC, LDL-C, eGFR, UA, antiplatelet drugs, statins, betablockers, ACEI/ARB, Oral hypoglycemic drugs, Insulin.

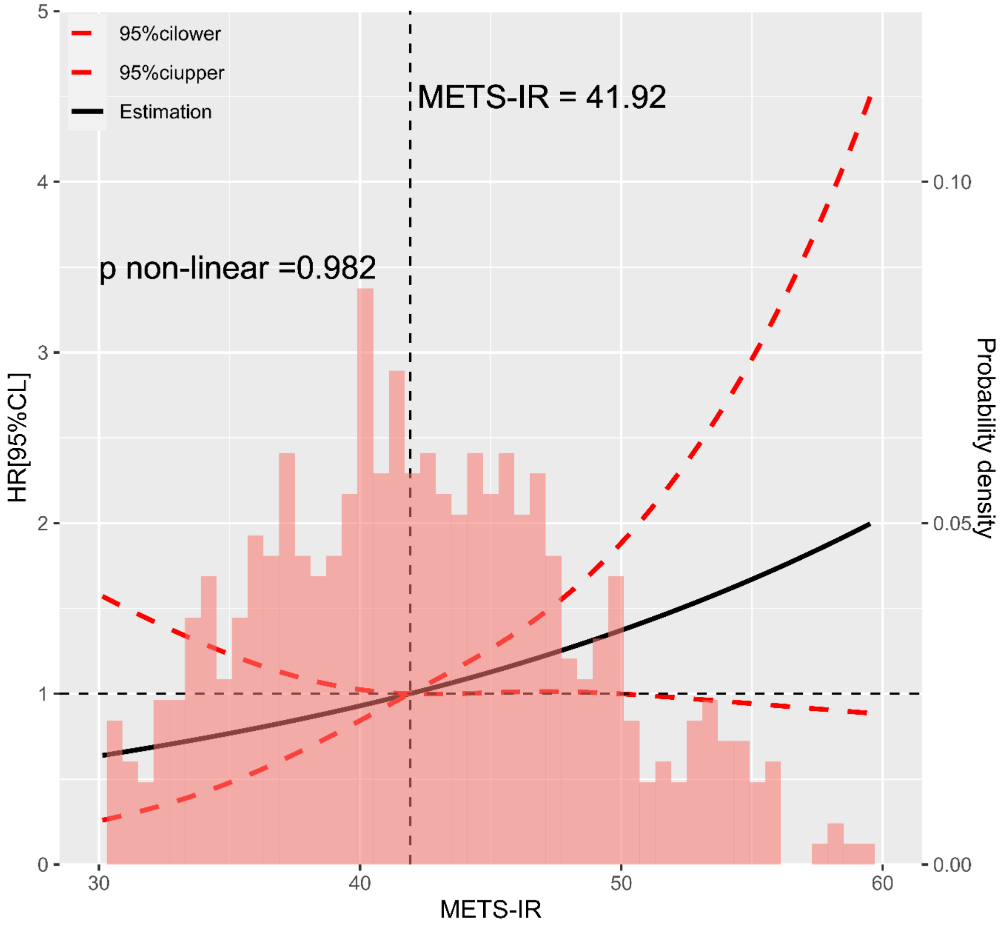

Supplementary Figure 2. The association between METS-IR and repeat coronary artery revascularization. Adjusted for age, gender, LVEF, admission for MI, multivessel disease, GS, current smoking, FH-CAD, DM, hypertension, TC, LDL-C, eGFR, UA, antiplatelet drugs, statins, betablockers, ACEI/ARB, Oral hypoglycemic drugs, Insulins.

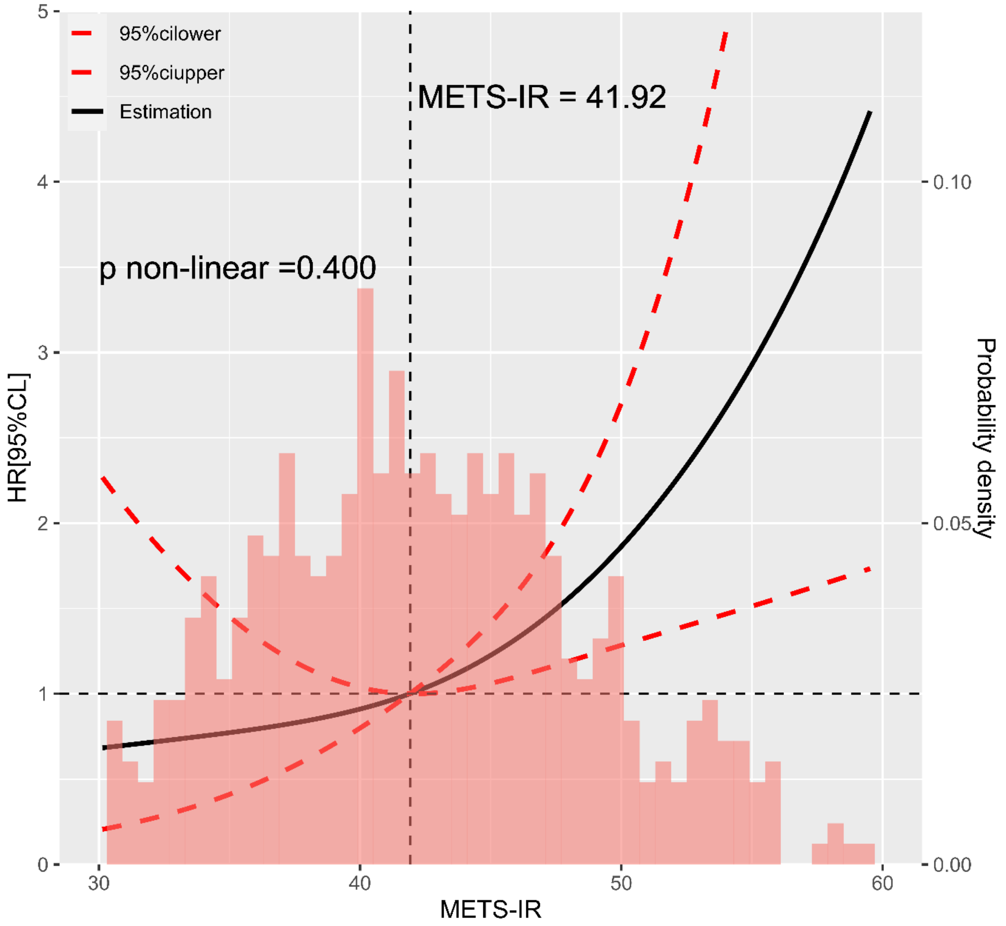

Supplementary Figure 3. The association between METS-IR and non-fatal MI. Adjusted for age, gender, LVEF, admission for MI, multivessel disease, GS, current smoking, FH-CAD, DM, hypertension, TC, LDL-C, eGFR, UA, antiplatelet drugs, statins, betablockers, ACEI/ARB, Oral hypoglycemic drugs, Insulins.
