# Supplementary material for: Metabolic score for insulin resistance predicts major adverse cardiovascular event in premature coronary artery disease
Source: Aging (Albany NY). 2024 Apr 1;16(7):6364–83. doi: 10.18632/aging.205710 (PMC11042949; doi:10.18632/aging.205710)
Supplement: Supplementary Tables [file aging-16-205710-s002.pdf]

SUPPLEMENTARY TABLES

Supplementary Table 1. Baseline characteristics of the study and lost population.

Variables	Study	Lost	*p*-value
**General conditions**			
Age (years)	44.43±6.33	44.62±6.56	0.406
Male, n (%)	352(60.5)	156 (58.6)	0.613
BMI (kg/m^2)	26.89±3.35	26.79±3.47	0.622
LVEF (%)	60.00(56.00-65.00)	60.00(57.00-65.00)	0.866
Admission for MI, n (%)	161(27.7)	79(29.7)	0.541
GS	36.75(21.00-61.00)	34.00(20.00-60.25)	0.692
Multivessel disease, n (%)	261(44.8)	114(42.9)	0.589
**Risk factors, n (%)**			
Current Smoking	172(29.6)	70(26.3)	0.333
FH-CAD	167(28.7)	73(27.4)	0.708
DM	124(21.3)	58(21.8)	0.870
Hypertension	321(55.2)	146(54.9)	0.942
**Laboratory test**			
FPG (mg/dL)	89.02(81.09-108.03)	89.11(81.43-111.90)	0.890
TC (mg/dL)	154.45(129.12-185.28)	154.64(130.19-189.82)	0.521
TG (mg/dL)	128.39(97.44-180.05)	126.62(97.40-175.18)	0.727
LDL-C (mg/dL)	95.10(74.13-118.40)	95.49(74.90-121.01)	0.673
HDL-C (mg/dL)	95.10(74.13-118.40)	40.98(35.57-48.71)	0.957
eGFR (mL/min/1.73m^2)	114.52(100.54-130.45)	114.38(102.12-131.04)	0.899
UA (µmol/L)	318.50(257.00-371.25)	315.50(254.75-367.00)	0.553
**Discharge medications, n (%)**			
Antiplatelet drugs	551(94.7)	251(94.4)	0.970
Stains	555(95.4)	252(94.7)	0.694
Beta-blockers	369(63.4)	165(62.0)	0.701
ACEI/ARB	265(45.5)	123(46.2)	0.848
Oral hypoglycemic drugs	66(11.3)	33(12.4)	0.654
Insulins	25(4.3)	14(5.3)	0.533
METS-IR	42.33±7.25	42.08±7.31	0.998

Data were given as mean ± SD, median with interquartile range or n (%).
MACE, major adverse cardiovascular events; BMI, body mass index; LVEF, left ventricle ejection fraction; MI, myocardial infarction; GS, Gensini score; FH-CAD, family history of coronary artery disease; DM, diabetes mellitus; FPG, fasting plasma glucose; TC, total cholesterol; TG, triglyceride; LDL-C, low-density lipoprotein-cholesterol; HDL-C, high-density lipoprotein-cholesterol; eGFR, estimated glomerular filtration rate; UA, uric acid; ACEI, angiotensin-converting enzyme inhibitors; ARB, angiotensin receptor blockers; METS-IR, metabolic score for insulin resistance.

Supplementary Table 2. Sensitivity analysis between METS-IR and endpoints.

METS-IR	HR (95%CL)		
	**MACE**	**Repeat coronary artery revascularization**	**Non-fatal MI**
Per 1 Unit increase	1.05(1.02-1.08) *	1.04(1.00-1.08) *	1.07(1.02-1.13) *
Per 1 SD increase	1.40(1.14-1.72) *	1.32(1.00-1.73) *	1.68(1.19-2.38) *
Tretile1	Reference	Reference	Reference
Tretile2	1.74(1.00-3.01) *	1.51(0.73-3.12)	2.47(0.90-6.80)
Tretile3	2.58(1.46-4.55) *	2.39(1.15-4.97) *	3.85(1.36-10.90) *
p for trend	**0.001**	**0.017**	**0.010**

Multivariable model adjusted for age, gender, LVEF, admission for MI, multivessel disease, GS, current smoking, FH-CAD, DM, hypertension, TC, LDL-C, eGFR, UA, antiplatelet drugs, statins, betablockers, ACEI/ARB, Oral hypoglycemic drugs, Insulins.

The sensitivity analysis excluded events occurring in first six months of follow-up.

MACE, major adverse cardiovascular events; METS-IR, metabolic score for insulin resistance; HR, hazard ratio; CI, confidence interval; SD, standard deviation; Non-fatal MI, non-fatal myocardial infraction.

p-values in bold are <0.05.

*p <0.05.
